# Primary Hyperaldosteronism in a Normotensive Patient: A Case Report

**DOI:** 10.5812/ijem-138703

**Published:** 2023-12-13

**Authors:** Amir Hossein Ghanooni, Mitra KazemiJahromi, Farhad Hosseinpanah

**Affiliations:** 1 Department of Endocrinology, School of Medicine, Iran University of Medical Sciences, Tehran, Iran; 2 Endocrinology and Metabolism Research Center, Hormozgan University of Medical Sciences, Bandar Abbas, Iran; 3 Obesity Research Center, Research Institute for Endocrine Science, Shahid Beheshti University of Medical Sciences, Tehran, Iran

**Keywords:** Primary Aldosteronism, Normotensive, Hypokalemia, Adrenal Tumor

## Abstract

**Introduction:**

Primary aldosteronism (PA) is a clinical syndrome characterized by hypertension, suppressed plasma renin activity (PRA), elevated plasma aldosterone concentration (PAC), and spontaneous hypokalemia.

**Case Presentation:**

We present a 37-year-old normotensive female with hypokalemia, high plasma aldosterone level, and suppressed renin. The patient was treated with eplerenone and potassium chloride supplement. Further investigation with a computed tomography (CT) scan revealed a mass in the left adrenal. Laparoscopic adrenalectomy led to the diagnosis of adrenal adenoma.

**Conclusions:**

Primary aldosteronism should be among the differential diagnoses in normotensive patients presenting with severe hypokalemia.

## 1. Introduction

Primary aldosteronism (PA) is an uncommon but very important disease characterized by hypertension, suppressed plasma renin activity (PRA), elevated plasma aldosterone concentration (PAC), and hypokalemia. Hypertension is an essential element for physicians to consider when considering the diagnosis of PA (1, 2), and most clinicians do not request PRA and PAC levels in normotensive patients, even in the presence of hypokalemia. According to the Endocrine Society clinical practice guideline for screening PA, patients with moderate to severe hypertension and/or resistant hypertension or patients with mild hypertension and hypokalemia should be screened for PA. Hence, there is no recommendation in this guideline to screen normotensive patients with severe hypokalemia who have no evidence of gastrointestinal loss for PA (2, 3). Moreover, the approach to adrenal incidentaloma in the American Association of Clinical Endocrinologists guideline recommends screening for PA only in the presence of hypertension (4).

Ito et al. reported evidence that primary aldosteronism is not confined to patients with moderate to severe and/or resistant hypertension but also exists in patients with mild hypertension and even in normotensive patients (5). Moreover, recent studies have reported more confirmed cases of PA with suppressed renin and normal blood pressure, highlighting a wider spectrum of PA (6). It appears that hypertension is confined to more advanced stages of renin-independent hyperaldosteronism and may represent only the “tip of the iceberg” (6).

In this paper, we report a case of normotensive PA with hypokalemia, elevated PAC, suppressed PRA, and adrenal mass.

## 2. Case Presentation

A 37-year-old female presented with hypokalemia following routine tests during follow-up after abdominal surgery (subsequent to the rupture of the diaphragm) 3 years before the final diagnosis. She had no history of any diseases and no specific diagnostic or therapeutic management except for high potassium intake in the first year after the detection of hypokalemia. She had muscle cramps and weakness, along with hypokalemia.

After a year, she was referred to a nephrologist because her weakness progressed, and she developed polyuria, nocturia, and a headache. She was admitted several times due to severe hypokalemia and weakness. During this period, she received the following medications to control hypokalemia: Intravenous potassium chloride and various oral medications, including spironolactone up to a maximum of 500 mg per day (discontinued due to menstrual disorders); eplerenone 100 mg twice daily; potassium chloride 600 mg tablets up to 5 doses per day. Within all outpatient and inpatient visits, the blood pressure was normal (Table 1). 

**Table 1.** Blood Pressure and Lab Findings of Patient from First to Last Visit Before Surgery

Variables	BP, Max/Min, (mmHg)	Cr, (mg/dL)	K^+^, (mEq/L)	Na^+^, (mEq/L)	Mg^++^, (mg/dL)	Protein in Urine, (mg/24h)	WBC(/µL), Hb(g/dL), Plt(/µL)	TSH, (µIU/mL)	VBG, PH, HCO_3_(mmol/L), PCO_2_(mmHg)	PAC (ng/dL)	PRA (ng/mL/h)
**First admission, (18/04/2015) ** ^**a**^	120/70	0.6	2.5	140	2.1	320,	7300, 11.8, 210000	1.93			
**Second admission, (30/04/2015)**	120/70		3.3 (TTKG = 15.3)	138							
**Third admission, (01/10/2015)**	120/70		3.1	143		672					
**Forth admission, (11/06/2016)**	130/90	0.6	3.3	137	2.3			2.2	7.47, 27.5, 38.3	39, (4 - 31)	N/A
**Final lab tests (before surgery), (22/06/2016)**	140/95	0.9	3	143						25, (2 - 18)	0.6, (0.2 - 1.6)

Abbreviation: N/A, not applicable; BP, blood pressure; Cr, creatinine; Hb, hemoglobin; K, potassium; Plt, platelet; Na, sodium; TSH, thyroid-stimulating hormone; TTKG, transtubular potassium gradient; VBG, venous blood gas; WBC, white blood count.

^a^ The first admission to correct and evaluation of hypokalemia was 2 years after detection of hypokalemia.

The family history of hypertension was negative. There was a history of diabetes mellitus in her mother, sister, and two brothers. There was a history of proteinuria in her mother (possibly diabetic nephropathy).

**Figure 1.**
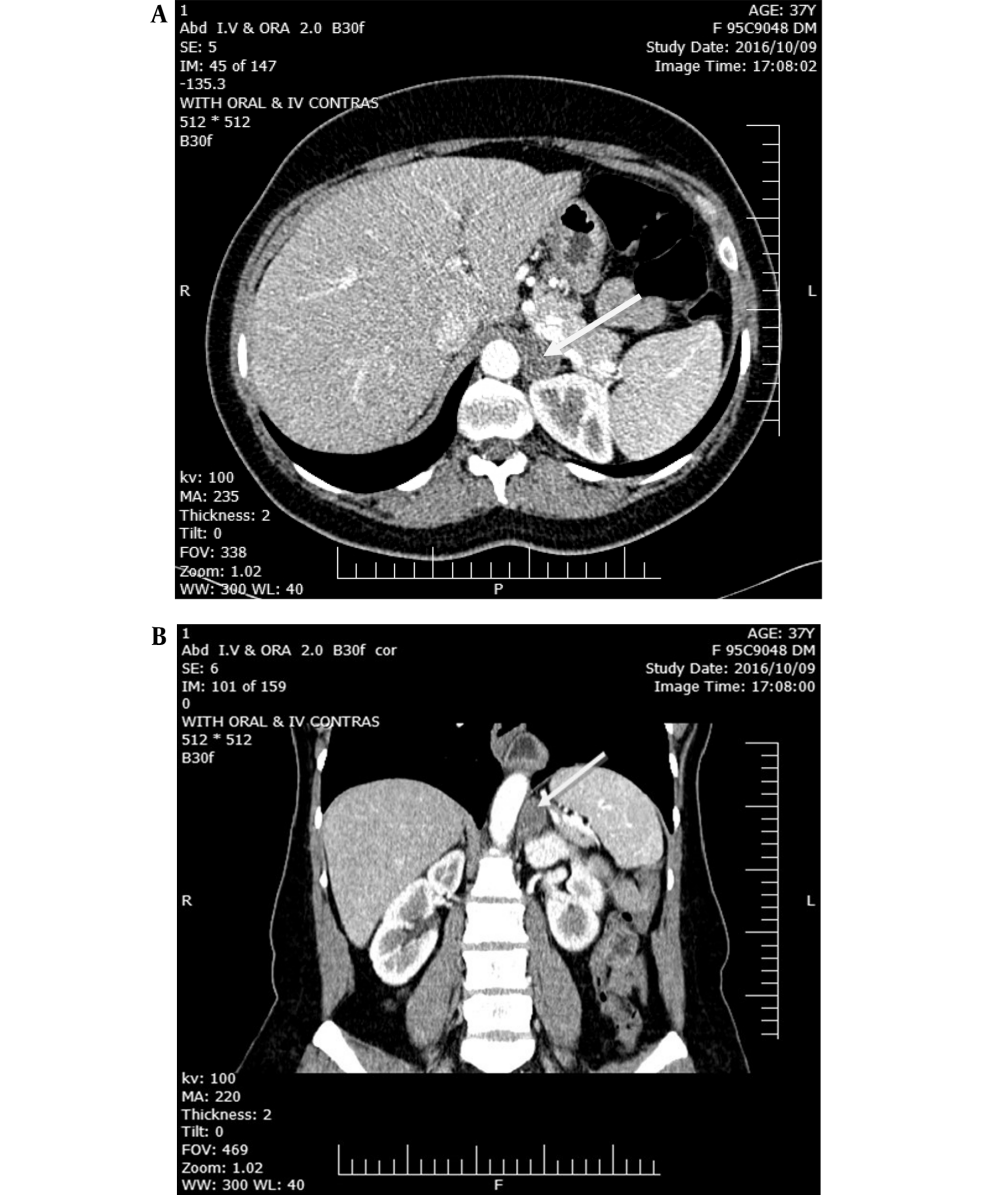
Left hypodense and homogenous adrenal mass (arrow) in transverse view (A) and coronal view (B).

Two years later, following a urinary tract infection (UTI), she was admitted to the hospital. A computed tomography scan was ordered to rule out complicated pyelonephritis. Incidentally, a homogenous, well-defined mass of 21 × 17 mm and Hounsfield Unit (HU) = - 10 was detected in the left adrenal (Figure 1). In an approach to the adrenal mass, PAC and PRA were requested after the treatment of hypokalemia (Table 1). It is worth mentioning that it was very difficult to discontinue eplerenone. The test was carried out while taking eplerenone. Considering the possible interference of eplerenone with the results of the aldosterone/renin ratio (ARR) -potassium-sparing diuretics increase the level of renin and then cause a false negative of ARR- therefore, the suppression of renin level along with the strongly positive ARR level in our patient (41.6 ng/dL/ng/mL/h) was considered a positive test, and no confirmatory tests were ordered. Accompanied by a history of hypokalemia and a well-defined adrenal mass, it was interpreted as a confirmed aldosterone-producing adenoma. Taken all together, we decided to recommend laparoscopic surgery.

Ultimately, laparoscopic surgery was performed, and a left adrenalectomy was done. The pathology report revealed adrenal adenoma. At the postoperative visit, hypokalemia was resolved without any medication, and blood pressure was 100/70 mmHg. In several visits after surgery, the average blood pressure of the patient was 95/60 mmHg, and potassium levels were still within the normal range without any medical treatment, even after 3 years.

## 3. Discussion

PA is the most common cause of secondary hypertension (1-3). Although the prevalence of normotensive PA is lower than PA with severe and resistant hypertension, a substantial number of patients with PA may be normotensive (5). According to a study by Ito et al., PA is not limited to patients with moderate to severe and/or resistant hypertension and can be seen even in those who are not at high risk for hypertension (5).

The prevalence of normotensive PA is probably underestimated because most patients with PA are normokalemic and/or are subsequently detected as adrenal incidentaloma. Normokalemic hypertension is the most common presentation of PA, and the occurrence of hypokalemia indicates advanced disease (2).

A normal blood pressure profile in PA indicates the presence of protective mechanisms against hypertension (7). After Brooks et al. introduced normotensive PA in 1972 (3), many studies have been conducted in this respect. These studies showed that the prevalence of normotensive PA was higher in the Oriental populations (especially Japanese) than in the Western populations, especially among women in an age range of 23 - 55 years, suggesting that genetic and gender-related protective factors play a role in response to hyperaldosteronism. However, no family cases have been reported (8, 9).

The mechanism of normal blood pressure in normotensive PA is still unclear. However, there are some explanations.

(1) Patients are still in the early stages of the disease. Hypertension might gradually develop over time after the decompensation of defensive mechanisms like vasodilation and/or Na^+^ wasting. Secretion or sensitivity of vasodilator substances, such as prostaglandin E, kallikrein, and nitric oxide, increases while the sensitivity of vasoconstrictor substances reduces (7).

(2) The previous blood pressure of patients is low and normal and remains within normal range even in hypertensive cases (10).

(3) The occurrence of the aldosterone escape phenomenon could be due to the inhibition of the endogenous renin-angiotensin-aldosterone system due to the role of genetic and environmental factors, thereby increasing sodium excretion and vasodilation (5, 10).

(4) A long-term low-sodium diet may help the patient to keep the blood pressure normal (7).

(5) A minority of patients with Bartter–Gitelman syndrome have normal blood pressure because of Na^+^ wasting (11).

The approach to our patient was based on the hypokalemia approach algorithm by nephrologists in the first place. According to the well-known approach algorithm to hypokalemia (12), diarrhea, vomiting, use of diuretics, Bartter's disease, Gitelman's disease, renal tubular acidosis, and diabetic ketoacidosis are included in the differential diagnosis of hypokalemia in a patient with normal blood pressure (12). In this algorithm, the evaluation of hyperaldosteronism in cases without hypertension is not recommended, so it seems that this patient was not evaluated for hyperaldosteronism for initial evaluation. In our patient, due to the incidental observation of an adrenal mass after prolonged hypokalemia, further investigations were directed toward primary hyperaldosteronism by endocrinologists. Very few similar cases have been introduced in the world in which primary hyperaldosteronism was present without high blood pressure. Usually, patients were diagnosed during workup for adrenal incidentaloma (13) or hypokalemia (9, 14-16).

In our patient, an adrenalectomy lowered blood pressure and corrected refractory hypokalemia without any medication. A significant decrease in blood pressure was observed after surgery. The rising trend of blood pressure levels from the first visit to the time of surgery shows that hypertension was gradually developing in our patient. Because at the onset of the disease, the patient's baseline blood pressure was in the low normal range, in the process of increasing her blood pressure, hypertension was developed after about three years.

Regarding the diagnosis of PA, it appears that we are at the end of a journey. The journey started with hypertension and hypokalemia, continued with hypertension and normokalemia, and ended with hypokalemia and without hypertension. Therefore, it is reasonable to consider PA as a possible diagnosis when physicians encounter a case of hypokalemia that is not accompanied by hypertension and gastrointestinal loss.
